# The predictive role of marital satisfaction on the parental agreement

**DOI:** 10.1002/nop2.571

**Published:** 2020-07-23

**Authors:** Zahra Vafaeenejad, Forouzan Elyasi, Mahmood Moosazadeh, Zohreh Shahhosseini

**Affiliations:** ^1^ Department of Reproductive Health and Midwifery Student Research Committee Mazandaran University of Medical Sciences Sari Iran; ^2^ Psychiatry and Behavioral Sciences Research Center Addiction Institute Mazandaran University of Medical Sciences Sari Iran; ^3^ Department of Psychiatry School of Medicine Sexual and Reproductive Health Research Center University of Medical Sciences Sari Iran; ^4^ Health Sciences Research Center Addiction Institute Mazandaran University of Medical Sciences Sari Iran; ^5^ Department of Reproductive Health and Midwifery Sexual and Reproductive Health Research Center Mazandaran University of Medical Sciences Sari Iran

**Keywords:** child‐rearing, co‐parenting, marital conflict, parenting, parenting styles

## Abstract

**Aim:**

The aim of this study was to investigate the predictive role of marital satisfaction on similarities in parenting styles.

**Design:**

The present cross‐sectional study was conducted on 617 Iranian father–mother dyads in 2018.

**Methods:**

During a systematic sampling method, Afrooz Marital Satisfaction Scale and Parenting Styles and Dimensions Questionnaire were administered. The data were analysed using a *t* test, a chi‐square test, one‐way ANOVA and logistic regression.

**Results:**

Results showed that 71.47% of the couples exhibited agreement in parenting styles and that 82.50% reared their children in an authoritative manner. The logistic regression indicated that increased marital satisfaction among fathers (AOR: 3.19; CI: 2.06, 4.92) and mothers (AOR: 2.74; CI: 1.76, 4.25) could elevate the odds of correspondence in parenting styles. The findings suggest that agreement on parenting styles should be considered when evaluating marital satisfaction in couples. Intervention targeting marital satisfaction to improve parental agreement is recommended.

## 1 INTRODUCTION

Family systems are regarded as dynamic social structures that are made up of mutually influencing sub‐systems, such as marital relationships, parent–child relationships and sibling relationships (Favez, Tissot, & Frascarolo, 2016; Korja et al., 2016). Within family systems, co‐parenting encompasses issues such as agreement or disagreement in parenting styles, which can affect child health (Schoppe‐Sullivan & Mangelsdorf, 2013; Teubert & Pinquart, 2010). Parenting style refers to the set of strategies adopted by parents to regulate the behaviours of their children (Yu & Gamble, 2008). It is classified into three major approaches, namely authoritative, authoritarian and permissive (Alavi, Afrooz, & Ghasemzadeh, 2015; Smetana, 2017). Agreement in parenting style refers to similarities in how couples bring up their children and correspondence with respect to the values and aspirations that underlie child‐rearing. Such agreement is an effective parenting criterion because it creates a stable familial environment and enables its optimal functioning (Hemmingsson, Ólafsdóttir, & Egilson, 2017; Jones et al., 2019; Winsler, Madigan, & Aquilino, 2005). It is also accompanied by desirable children's health outcomes, such as reduced behavioural problems and improved psychological functioning (Don, Biehle, & Mickelson, 2013; Rinaldi & Howe, 2012; Teubert & Pinquart, 2010).

The family system perspective further suggests that the marital relationship between mothers and fathers plays an important role in children's psychological health, particularly with regard to how it may affect parents' child‐rearing practices (Bortz, Berrigan, VanBergen, & Gavazzi, 2019; Schoppe, Mangelsdorf, & Frosch, 2001; Winsler et al., 2005). For an explanation of this issue, some theories and hypotheses were defined. Emotional security theory is a famous theory in this era (Davies, Martin, & Sturge‐Apple, 2016; López‐Larrosa, Sánchez‐Souto, Ha, & Cummings, 2019), that implies when a child is exposed to destructive parental conflict and subsequent disagreement in parenting style, this leads to feelings of emotional insecurity, which increases the risk of child psychological problems. The other explanations are existing by considering attachment theory and the spillover hypothesis as well as the compensatory hypothesis. The spillover hypothesis suggests that effect or behaviour transfers directly from one setting or relationship to another within a family system and it is anticipated that marital conflict is expressed in the form of inappropriate parenting (Martin, Sturge‐Apple, Davies, Romero, & Buckholz, 2017). On the other hand, the hypothesis of compensation is that people try to compensate for a defect in one system by trying to improve it in another system and thus parents with poor marital satisfaction try to compensate it by improving their relationships with children (Hollist & Miller, 2005). These evidences show studies in parents' relationships and subsequent parenting styles are important.

Although prior research has established the relationship between marital satisfaction and parenting style (Alavi et al., 2015; Camisasca, Miragoli, Di Blasio, & Feinberg, 2019; Ponnet et al., 2013), little is known about influences of marital satisfaction on parenting agreement (Margolin, Gordis, & John, 2001; Pedro, Ribeiro, & Shelton, 2012; Schoppe‐Sullivan & Mangelsdorf, 2013). Indeed, studies in this area to date have been an almost exclusive focus on one of the parents and in the most cases relationship of mothers' marital satisfaction with parenting style (Chang, Lansford, Schwartz, & Farver, 2004; Haghighi & Khalilzadeh, 2012; Yu & Gamble, 2008). The practical importance of these findings is by investigation the related factors to parental agreement and due to the critical role of parental agreement on children's' well‐being, we can learn more about the interventions needed to promote parental agreement in families.

The aim of this study was to investigate the predictive role of marital satisfaction parenting styles of both parents. We intended to answer this question "Do couples with marital satisfaction exhibit agreement in parenting styles?"

## 2 METHOD

### 2.1 Study setting and participants

In this descriptive correlational research, during systematic sampling method 617 couples from household in two primary healthcare centres in Minudasht City, north‐eastern Iran, were recruited. At first, a telephone‐based invitation was sent to the participants. They were requested to attend the above healthcare centres to complete self‐administrated questionnaires. The inclusion criteria were parents with a child between the ages of 6–12 years old, living in a two‐parent familial structure, writing and reading literacy, monogamy of husband and willingness to participate in the study. The exclusion criteria included no completion of the questionnaires by at least one of the parents. Data gathering was conducted between mid‐July–end‐December 2018.

Conformity to a code of ethics was ensured by obtaining ethical approval from the Ethics Committee of the Vice‐Chancellor's Office for Research at Mazandaran University of Medical Sciences (Ethical code: IR.MAZUMS.REC. 2942), securing written consent forms from the participants and ensuring the confidentiality of collected information.

### 2.2 Sample size

The sample size was determined to be 678 subjects with a 99% confidence level (*α* = 0.01), 90% power (*β* = 0.01), design effect = 1.5 and *r* = −.18 correlation coefficient between marital satisfaction and parenting style reported in a former study (Haghighi & Khalilzadeh, 2012) using G Power software. Finally, 617 Iranian father–mother dyads completed the questionnaires (response rate = 91%).

### 2.3 Measures

For evaluating marital satisfaction, Afrooz Marital Satisfaction Scale was administrated. It was developed and validated by Ghodrati, Afroz, Sharifi, and Homan (2011) in the Iranian context. This culturally sensitive scale comprises 51 items on a five‐point Likert scale (“never,” “once in a while,” “about half of the time,” “very often,” “always”). Scores range from 51–204. Parents with scores less than 175 are regarded as having no marital satisfaction. Cronbach's *α* = 0.95 was reported in terms of reliability of this scale. Also, its consistency was established with test–retest reliability and intra‐cluster correlation coefficient = 0.79, *p* < .001, with an interval of 20 days. Its concurrent validity with Enrich's marital satisfaction questionnaire = 0.43, *p* < .001 indicated an appropriate reliability of this scale as well (Ghodrati et al., 2011; Ghodrati‐Ali & Ghodrati, 2011).

Parenting Styles and Dimensions Questionnaire (PSDQ) was used to evaluate parenting styles. Robinson et al. developed the PSDQ, which contains 32 items that includes three categories of parenting styles: 15 items related to authoritative parenting, 12 items associated with authoritarian parenting and five items concerning permissive parenting. Each item is evaluated on a five‐point Likert scale (“never,” “once in a while,” “about half of the time,” “very often,” “always”) (Olivari, Tagliabue, & Confalonieri, 2013). The internal consistency values of the scale, as determined on the basis of Cronbach's *α*, were 0.64, 0.86 and 0.82 for authoritative, authoritarian and permissive parenting styles, respectively (Morowatisharifabad et al., 2016; Rinaldi & Howe, 2012). In this study, scores related to parenting styles were determined using the PSDQ with a high score for the different styles reflecting the dominant approach espoused by a respondent (Howenstein et al., 2015). To investigate agreement in parenting styles between the participating couples, the way they each bring up their children was compared. Couples with the same parenting style were assigned a code of 1, which denotes agreement in styles and those with dissimilar parenting styles were ascribed a code of 2, which indicates disagreement in the manner of rearing children (Komijani & Maher, 2007). The socio‐demographic checklist comprises items regarding age, educational level, employment status, number of children, duration of marriage and perceived socio‐economic status.

### 2.4 Statistical analyses

The collected data were entered into the Statistical Package for the Social Sciences (version 16.0, SPSS Inc., Chicago, Illinois, USA). Mean and standard deviation were used to describe quantitative variables, whereas number and percentage were employed to illustrate qualitative ones. A *t* test and a chi‐square test were conducted to determine the relationship between the variables. Finally, logistic regression was used to predict the odds of being marital satisfaction based on the value of the independent variable (similarities in parenting styles).

## 3 RESULTS

The mean ages of the mothers and fathers were 34.78 (*SD* 5.48) and 38.50 (*SD* 16.64) years, respectively. The other characteristics of the participants are summarized in Table 1. Among the parents, 441(71.47%) exhibited agreement in parenting styles and 82.50% raised their children in an authoritative fashion. Authoritarian and permissive parenting styles were adopted by only 10.90% and 6.60% of the respondents, respectively (Table 2).

**TABLE 1:** Demographic characteristics of participants

Variable	Mothers (*N* = 617)	Fathers (*N* = 617)
Age^a^	34.78 (5.48)	38.50 (16.64)
Number of children^a^	2.20 (8.35)	2.20 (8.35)
Duration of marriage^a^	13.54 (4.80)	13.54 (4.80)
Marital satisfaction^a^	172.00 (24.48)	175.91 (23.40)
Educational level^b^		
Primary	179 (29.00)	185 (30.00)
Secondary	271 (43.90)	226 (36.60)
Higher education	167 (27.10)	206 (33.40)
Employment status^b^		
Employed	118 (19.10)	592 (95.90)
Unemployed	449 (80.90)	25 (4.10)
Perceived socio‐economic statues^b^		
High	245 (39.70)	223 (37.80)
Moderate	310 (50.20)	306 (49.60)
Low	62 (10.10)	78 (12.60)
Parenting style^a^		
Authoritative (Range: 15–75)	53.18. (13.18)	53.55 (13.55)
Authoritarian (Range: 12–60)	26.07 (7.53)	25.39 (7.77)
Permissive (Range: 5–25)	11.41 (3.52)	11.15 (3.35)

^a^ Mean (*SD*).

^b^ Frequency (per cent).

**TABLE 2:** Frequency (per cent) of parenting styles in participants

Parenting style	Mother (*N* = 617)	Mother (*N* = 617)	Both parents (*N* = 441)^a^
Authoritative	427 (69.2)	441 (71.05)	364 (82.50)
Authoritarian	103 (16.7)	101 (16.4)	48 (10.90)
Permissive	87 (14.1)	75 (12.2)	29 (6.60)

^a^ Among those who have parental agreement (*N* = 441).

The results showed the mean score of couples' marital satisfaction is different based on agreement in parenting styles (*p* < .001). In this way, the highest (183.43 ± 18.75) and the lowest (147.41 ± 16.46) scores of mothers' marital satisfaction score were found when they adopted authoritative and authoritarian parenting style, respectively (*p* < .001). Accordingly, the highest fathers' marital satisfaction (187.78 *SD* 16.63) was found when both of parents were authoritarianism and the lowest fathers' marital satisfaction score (148.64 *SD* 15.01) was reported when they were authoritativeness (*p* < .001) (Table 3). The logistic regression showed that increased marital satisfaction among fathers (AOR: 3.19; CI: 2.06, 4.92) and mothers (AOR: 2.74; CI: 1.76, 4.25) could increase the odds that parenting styles will agree (Table 4).

**TABLE 3:** Mean (standard deviation) of couples' marital satisfaction score based on their agreement in parenting styles

Couples' agreement in parenting style	Both authoritative (*N* = 364)	Both authoritarian (*N* = 48)	Both permissive (*N* = 29)	No agreement (*N* = 176)	*p*
Mothers' marital satisfaction	183.43 (18.75)	147.41 (16.46)	154.89 (14.87)	157.87 (24.21)	<.001
Fathers' marital satisfaction	187.78 (16.63)	148.64 (15.01)	153.65 (14.65)	162.45 (22.31)	<.001

**TABLE 4:** Summary of regression models of the parental agreement, based on marital satisfaction

Variables	Univariate analysis		Bivariate analysis	
	Odds ratio (CI 95%)	*p*	Adjusted odds ratio (CI 95%)	*p*
Mothers' marital satisfaction	4.90 (3.34–7.27)	<.001	2.74 (1.76–4.25)	<.001
Father's marital satisfaction	5.29 (3.62–7.73)	<.001	3.19 (2.06–4.92)	<.001

## 4 DISCUSSION

Family as a dynamic social system is influenced by communication between family members, and mothers' and fathers' parenting styles are conceptualized as being interdependent (Korja et al., 2016; Van Auken & Werbel, 2006). So, the relationship between mothers' and fathers' parenting makes an important contribution to children's functioning, perhaps more important than the individual contributions of mothers' or fathers' parenting style (Chen & Johnston, 2012; Don et al., 2013) and research around this issue can promote limited literature on this issue.

The results indicated the marital satisfaction is a predictive factor for increasing the odds of correspondence in parenting styles. These results align with some studies reported parents with similar parenting styles experienced minimal conflict in their marital relationships (Harvey, 2000; Margolin et al., 2001). It seems in satisfactory marital relationships, good feelings about the spouse can lead to better interaction between parents and can cause parents to talk more about their children and, as a result, lead to more engagement in parenting styles agreement (Boričević Maršanić & Kušmić, 2013; Margolin et al., 2001). The higher odds ratio of the predictive role of fathers' marital satisfaction on the parental agreement (3.19), compared with mothers (2.74), could be due to the Iranian patriarchal society (Zaheri et al., 2016). This finding could help we better understand the impact of marital relationships on parenting styles in Iranian society and developing interventional studies targeting fathers' marital satisfaction in the future.

Our results suggest that higher marital satisfaction score is associated with authoritative parenting style and vice versa when parents adopted authoritarian parenting style, the marital satisfaction score was at least (*p* < .001). It is speculated couples with satisfactory marital relationships can transfer the desirable feelings that they have for their spouses to parental interactions, thereby engendering positive parental interactions (Harvey, 2000; Margolin et al., 2001).

Most parents participating in this research had congruent parenting styles in terms of authoritative parenting. In line with results of this study, it is showed parents who had high levels of agreement in parenting styles were those who adopted positive parenting, whereas those exhibiting low agreement in parenting approaches were couples who implemented ineffective parenting (Kuppens & Ceulemans, 2019). As in line with other studies (Komijani & Maher, 2007; Winsler et al., 2005), most parents participating in this research had authoritative parenting styles, and this congruent is anticipated.

### 4.1 Strengths and limitations

The current study contributes to the literature by addressing an important yet relatively neglected area of research. That is, the findings here do suggest that both mothers' and fathers' parenting styles need attention in future researches. The current study also points to some important directions for future research, specifically an examination of the factors influencing parental agreement on child‐rearing practices. The relatively large sample size (617 parents) of this project strengthened the power of the analyses that may make our conclusions and implications more inclusive.

However, the current study's findings should be considered in light of its limitations. First, the probability of the temporality bias must be considered as causal directions of relations among variables examined cannot be empirically evaluated because this study is cross‐sectional. Second, only self‐reported paper‐and‐pencil questionnaires were used to data gathering that may prone the result to the social desirability bias. Further randomized experiments can shed light on these matters by observing in‐home parenting behaviours and assessing the effects of interventions related to marital satisfaction in correspondence of parental agreement. Third, the generalizability of the findings from this study may be limited due to a restricted sample that was recruited from the primary healthcare centres and was relatively homogeneous in terms of socio‐economic status and geographic residence and is not representative of the more general population parents. Finally, no child outcome measures were available in the current work. It is clear that the next step could be a research programme to examine the mediating role of parental agreement on child health in couples with marital satisfaction.

## 5 CONCLUSION

In general, satisfactory marital relationships play an important role in the optimal functioning of parents as they raise their children. These factors can also dramatically pave the way for parental cooperation and agreement in adopted approaches to child‐rearing. The higher odds of parental agreement in couples' with more marital satisfaction illustrate the need to consider marital satisfaction as predisposing variables when investigating the co‐parenting and, in particular, point to the necessity to implement intervention programmes intended to promote couples' marital satisfaction.

## CONFLICT OF INTEREST

The authors declare that they have no conflict of interest.

## AUTHORS' CONTRIBUTION

All authors made a substantial contribution to writing of the paper draft and met the four criteria for authorship recommended by the International Committee of Medical Journal Editors.
